# Capturing patients, missing inequities: Data standardization on sexual orientation and gender identity across unequal clinical contexts

**DOI:** 10.1016/j.socscimed.2021.114295

**Published:** 2021-08-04

**Authors:** Taylor M. Cruz, Emily Allen Paine

**Affiliations:** a California State University, Fullerton, Department of Sociology, 2600 Nutwood Avenue, College Park 900, Fullerton, CA 92831, United States; b Columbia University and New York State Psychiatric Institute, HIV Center for Clinical and Behavioral Studies, 722 W 168th Street, New York, NY 10032, United States

**Keywords:** Electronic health records, Data analytics, Social determinants, Health inequity, Gender and sexual minority, LGBTQ health, Clinical settings, United States

## Abstract

In effort to address fundamental causes and reduce health disparities, public programs increasingly mandate sites of care to capture patient data on social and behavioral domains within Electronic Health Records (EHRs). Data reporting drawing from EHRs plays an essential role in public management of social problems, and data on social factors are commonly cited as foundational for eliminating health inequities. Yet one major shortcoming of these data-centered initiatives is their limited attention to social context, including the institutional conditions of biomedical stratification and variation of care provision across clinical settings. In this article, we leverage comparative fieldwork to examine provider and system responses to mandated data collection on patient sexual orientation and gender identity (SOGI), highlighting unequal clinical contexts as they appear across a large county safety-net institution and an LGBTQ-oriented health organization. Although point of care data collection is commonly justified for governance in the aggregate (e.g., disparity monitoring), we find standardized data on social domains presents a double-edged sword in clinical settings: formal categories promote visibility where certain issues remain hidden, yet constrain clinical utility in sites with greater knowledge and experience with related topics. We further illustrate how data standardization captures patient identities yet fundamentally misses these unequal contexts, resulting in limited attenuation of inequity despite broad expectations of clinical change. By revealing the often-invisible contexts of care that elude standard measurement, our findings underline the strengths of qualitative social science in accounting for the complex dynamics of enduring social problems. We call for deeper engagement with the unequal contexts of biomedical stratification, especially in light of increasing pressure to quantify the social amidst the rising tide of data-driven care.

Electronic Health Records (EHRs) and expanded data infrastructures have unleashed a new paradigm of “data-driven care” – comprising the incorporation of real-time data sources into clinical, administrative, and organizational decision-making within biomedicine – reflecting the continued technoscientific transformation of United States health care (Clarke et al., 2003; Ferris, 2010; Atasoy et al., 2019). New data sources on clinical encounters, patient populations, and system proceedings inform a range of high-tech innovations such as data dashboards and clinical algorithms (Galetsi et al., 2019; Chen et al., 2019; Obermeyer et al. 2019), with these same data infrastructures further linking clinical settings to state administration via mandated data reporting. Data reporting is essential for governance of health-related social problems, such as redressing social determinants and health disparities, and such data aggregate information across sites of care to publicize knowledge on related health domains (e.g., data.cms.gov, bphc.hrsa.gov/datareporting). The COVID-19 pandemic has only underscored the centrality of EHR infrastructure for societal management of social problems, with public pandemic response informed by real-time clinical process and outcome data drawing from mandated data reporting (e.g., covid.cdc.gov/covid-data-tracker).

One key dimension of data-centered management of social problems is the expansion of clinical data collection on patient social and behavioral domains within EHRs (also referred to as social determinants, social factors, and social risks and needs; Institute of Medicine, 2013; Cantor and Thorpe, 2018; NASDOH 2019). Reflecting a general desire to integrate medical care with broader movements towards redressing inequity, policy and scholarly audiences have called for EHR data collection on “social factors” such as housing and food insecurity, education, employment, and demographics such as race, ethnicity, and language (REAL) and sexual orientation and gender identity (SOGI) (Institute of Medicine, 2013; Douglas et al., 2015; Wasserman et al., 2019, Table 1). Calls for standardized social determinant data suggest data availability may induce providers and staff to address fundamental causes and reduce disparities directly within clinical settings (Institute of Medicine, 2014: 17; Douglas et al., 2015; Zhang et al., 2017). To address health inequities associated with gender and sexuality, for example, the Health Resources and Services Administration recently expanded US health center program requirements to include mandated data reporting on SOGI (HRSA 2016, Table 2). Standard data on SOGI within EHRs is then expected to help care teams identify patient-level tailored interventions while promoting cultural understanding of social differences, thereby supporting disparity reduction via data standardization (Cahill and Makadon, 2013; Fenway Health, 2015; Tables 1 and 2).

Despite their promise, these data-centered initiatives typically reflect limited grasp of the social context informing health inequities, including the institutional conditions of biomedical stratification as well as variation of care provision across clinical settings. In this article, we leverage qualitative fieldwork to compare provider and system responses to data reporting on “social factors” – using SOGI data as a case example – across a large county safety-net institution and an LGBTQ-oriented health organization. We find standard data on SOGI presents a double-edged sword within clinical settings: formal categories promote visibility in a context where sexual and gender minority (SGM) populations remain hidden, yet constrain clinical utility in a site with greater knowledge and experience with such patients. We also illustrate how data standardization captures patient identities yet fundamentally misses these unequal contexts, resulting in limited attenuation of inequity despite broad expectations of clinical change (Timmermans and Berg, 2003; Timmermans and Mauck, 2005). By revealing the often-invisible contexts of care that elude standard measurement, our findings underline the strengths of qualitative social science in accounting for the complex dynamics of enduring social problems. We call for scholarly, policy, and advocacy audiences to engage with and attend to the unequal contexts of biomedical stratification, especially in light of increasing pressure to quantify the social amidst the rising tide of data-driven care.

## 1. Background

Recent years have witnessed renewed scholarly and policy attention to redressing social factors within biomedicine, especially in relating social determinants and health disparities to practices of clinical care (Braveman et al., 2011; Cantor and Thorpe, 2018; NASDOH 2019). Although this work broadly references Link and Phelan (2010) classic conception of fundamental causes—outlined as social differences in flexible resources that allow certain people to gain health advantages more easily than others, emphasizing social conditions beyond the scope of care—more recent scholarship and policymaking frames clinical settings as sites wherein inequity is exacerbated or ameliorated (Shim, 2010; Starfield et al., 2012; Lutfey Spencer and Grace, 2016). Growing scrutiny of health system proceedings has in turn fueled a slew of EHR-based, data-centered initiatives, including expanded data reporting and public metrics tracking progress towards health equity (Penman-Aguilar et al., 2016; Anderson et al., 2018; DeMeester et al., 2017; Wasserman et al., 2019). Because standard data may lend visibility to otherwise hidden relations and facilitate population comparison across time and space, quantifying previously ignored “social factors” is framed to be key to redressing inequity. Advisory bodies have therefore recommended clinical data collection on social domains within EHRs, citing such data as foundational for eliminating inequities through care (Institute of Medicine, 2013; 2014; Adler and Stead, 2015, Table 1). EHR patient data on education, employment, and other demographics may allow providers and staff to apply point of care interventions to improve health outcomes, thereby linking clinical practice to desired objectives of disparity reduction.

The primacy of such data for reducing health inequities is particularly apparent within the realm of sexual and gender minority (SGM) health, given recognized “LGBTQ invisibility” within health care as well as lack of biomedical knowledge on population health (e.g., clinical implications of hormone use). In this article, we use “sexual and gender minority” (SGM) to describe diverse social domains and experiences that do not exclusively align with heterosexual or cisgender expectations. Similarly, we use “heteronormative” and “cisnormative” to characterize situations in which all people are assumed to be heterosexual and cisgender (i.e., based on dominant norms that privilege certain people and experiences while disadvantaging others).

According to HRSA, by collecting standardized SOGI data as part of health center reporting requirements (Table 2), providers may “improve the health of the nation’s underserved communities and vulnerable populations by assuring access to comprehensive, culturally competent, quality primary health care services… gaining a better understanding of populations served by health centers, including SOGI, promotes culturally competent care delivery and contributes to reducing health disparities overall” (HRSA 2016). Capturing SOGI data may further facilitate population-tailored care for SGM patients, such as recommending sexual minority men for HIV testing, or referring transgender people to endocrinologists for hormone replacement therapy (Institute of Medicine, 2013; Cahill and Makadon, 2013). Scholars, advocates, and public officials suggest including SOGI within EHRs via data reporting has been one of the most important interventions in reducing SGM health inequities, with data serving as the foundation for understanding population needs while promoting general visibility within biomedicine (Cahill and Makadon, 2013; Fenway Health, 2015).

Despite these well-meaning efforts to redress health inequities, data-centered approaches typically focus on *capturing patients* while missing the *unequal contexts* that sustain inequities within the health care landscape. Social scientists, in contrast, have highlighted the embeddedness of heteronormativity within biomedicine, as reflected in curricular priorities within health professions education, the demographic makeup of medical staff, and organizational segregation between sites of care (Cruz, 2014, 2020). Medical students’ uneven basic knowledge of SOGI (such as the distinction between SO and GI, and the multiple dimensions of identity, behavior, and embodiment) and low levels of comfort interacting with SGM patients is due in part to the absence of such topics within formal curricula (Obedin-Maliver et al., 2011; Carabez et al., 2015; Donald et al., 2017; Banerjee et al., 2018). Negative experiences in medical school, including implicit and explicit bias contributing to provider concealment of their own SGM identities, may inform future care disparities when providers enter the workforce (Wittlin et al., 2019; Phelan et al., 2017; Mansh et al., 2015). Lack of support for SGMs within medical institutions thus cannot be reduced to patient care practices alone: organizational segregation between LGBTQ-oriented clinics and majority health systems remains testament to institutional neglect in caring for diverse patients across all clinical settings (Ingraham and Rodriguez, 2021; Hadland et al., 2016; Martos et al., 2017). The implementation of evidence-based practices for certain sub-populations may be further undermined by limited institutional support, generating tensions between data-centered evidence and care capacity constraining even SGM-knowledgeable providers within patient care (Gaspar et al., 2020; Thompson, 2020). SGM patients also report limited understanding among medical staff *within* LGBTQ organizations, especially for transgender and nonbinary patients and those with multiple marginalized identities (Paine, 2018, 2021a, 2021b).

These institutional conditions powerfully shape SGM health inequities, creating unequal contexts across the stratified health care landscape. Such conditions, however, have attracted limited attention relative to data-centered approaches, with data collection within EHRs appearing as the dominant means of redressing such “social factors” (Cahill and Makadon, 2013; Fenway Health, 2015). There is good reason to suspect, however, that data programs—including HRSA’s recent expansion of SOGI data reporting—result in limited attenuation of health inequities: first year HRSA program results reported high amounts of missing data, with three-quarters and two-thirds missing for SO and GI respectively across all US health centers (Grasso et al., 2019). Advocates suggest these results may be attributed to poor implementation, yet this attribution provides little insight into more essential issues at hand, such as how the institutional conditions above influence provider and system responses to SOGI data collection. Our comparative fieldwork grounded in qualitative social science methods, in contrast, reveals how the social context of inequality varies considerably across sites of care, and how data-centered approaches insufficiently capture these unequal clinical contexts.

## 2. Data and methods

This article emerged as a collaborative project out of two independent studies. Though neither author initially set out to study the SOGI data initiative, both were immersed within two very different sites of care upon its standard rollout. The first author’s study examined the integration of data analytics within clinical care, comprising interviews and observations at a large county safety-net health system primarily serving low-income patients insured through Medicaid managed care (September 2017 to June 2018); the second author studied multilevel barriers and facilitators to SGM patient care at a LGBTQ-oriented health organization through qualitative fieldwork (October 2016 to August 2017). Given our respective research interests in SGM health inequities, both authors independently responded to this emergent phenomenon in the field by modifying our interview guides to include questions on the SOGI initiative. An impromptu conversation about our projects at the American Sociological Association’s 2019 meeting revealed our field sites had very different reactions to the mandate, motivating the present article.

**County Health System (Field Site 1)** is a large public integrated delivery system on the West Coast, comprising one main hospital, multiple outpatient clinics, a specialty center, and several ancillary services distributed through the region. As the county’s medical safety-net within the area’s stratified ecology of health services, patients, providers, and staff all exhibited a high level of racial, ethnic, and linguistic diversity, with many also reporting histories of immigration. As a public institution, the organization carries a mission of serving all patients without consideration of ability to pay. Despite this stated mission and urban geographic location, the health system rarely acknowledged SGM issues within its public messaging of care for “all.” The announcement of the SOGI mandate appeared within the organization’s existing data-centered accountable care initiatives, with the data understood as a new demographic reporting requirement following growing societal awareness of gender and sexuality.

**LGBTQ Health Center (Field Site 2)** is an LGBTQ health care center based out of the East Coast. Because few such sites exist in the US (Martos et al., 2017), we withhold some details to protect participant confidentiality. The site was characterized by a strong advocacy ethos, explicitly naming LGBTQ patients as intended recipients of care and centering attention towards SGM inequities within organizational initiatives. Patient-facing documents, such as intake forms and lobby messaging, contained language expressly recognizing sexual and gender diversity, and educational videos and posters also featured people whom the second author recognized to be LGBTQ. Providers and staff appeared to comprise primarily of SGM people, including white sexual minorities in leadership roles and a racially diverse frontline staff. Staff expressed a strong commitment towards caring for LGBTQ people as a way to redress inequity, and many were already attuned to issues of EHR data and SGM care, yet organizational concepts about sexuality and gender created ambivalence about capturing SOGI data following the reporting mandate.

### 2.1. Data analysis

Both projects drew from earlier analyses, represented as independent publications on SGM health inequities (Paine, 2018, 2021a, 2021b; Cruz, 2020). The first author’s project on data analytics within safety-net care was not originally designed to explore questions of gender and sexuality: following the principles of constructivist grounded theory (Charmaz, 2007), guided attention to SOGI data instead emerged in response to observed conflicts among providers and staff. The second author, in contrast, initiated the research specifically with gender and sexual inequities in mind, employing qualitative abductive analysis (Tavory and Timmermans, 2014) in targeting an LGBTQ health organization and drawing on existing literature prior to data collection. Despite these differences in methodology, both authors conducted fieldwork as trained medical sociologists and SGM people themselves. In initiating the collaboration, each author first returned to their own interview data and field notes, coding passages relevant to current research questions on SOGI data. We then presented these coded passages to each other, collaboratively discussing refined codes for further analyses and interpreting potential points of convergence across sites. After recoding our data and drafting analytical memos, we then exchanged drafts to finalize major axes of comparison across our two datasets. We also reflected on our own experiences conducting embedded fieldwork as SGM people within each organizational context, using extended conversation and draft exchange to draw final points of comparison across sites.

## 3. Findings

Table 3 summarizes our comparative findings, which we present through three social lenses: data as *formalized awareness*, with SOGI opening up attention to SGM patients while checkboxing diverse issues via standard categories; data as *bounded appropriateness*, with SOGI seen as the limit of acceptability versus a poor starting point for discussing gender and sexuality; and data as *limited information*, with SOGI failing to stand in for acquired knowledge and experience with SGM issues. We show how data standardization simultaneously captures patient identities while fundamentally missing unequal clinical contexts, presented through a systematic comparison of similarities and differences across sites.

### 3.1. Data as formalized awareness

As recipients of public funding, both field sites faced mandated SOGI data as a part of HRSA reporting requirements and disparity reduction initiatives. Both sites understood SOGI data as *formalized awareness* to issues of gender and sexuality, yet the formality engendered by standard data took on different meanings across sites: “opening up” gender and sexual diversity at County Health System, while “checkboxing” diverse issues at LGBTQ Health Center.

**At County Health System**, providers and staff confronted the data mandate in an environment where SGM issues were rarely given sustained organizational attention. This absence of formal consideration fueled an “LGBTQ invisibility” within clinical encounters and among providers and staff, resulting in a default heteronormativity extended to patients. Mandated SOGI data at this site thus drew attention to marginalized forms of gender and sexuality, adding the item to meeting agendas and provoking partial recognition among medical staff. In describing this formalized awareness, one complex care registered nurse (CCRN) drew a parallel between tobacco screening and SOGI – both previously “ignored,” yet now confronted via data mandates:

[Data collection] gives us more information so we are ultimately able to take better care of people. For example, the questionnaire item, “Do you use tobacco?” – even with a yes or no, we’re able to provide counseling, whereas before that question was completely ignored. And we know tobacco is a major health issue, so that has really opened the door for us to talk to patients, and without being judgmental. And now it’s coming, I see it coming similarly with SOGI. We are going to have to become more aware of those topics now. Because providers and staff … they’re not really used to those topics, to asking those kinds of questions.

In connecting data collection on SOGI to questionnaire items on tobacco use, this CCRN suggests similarities in how “social risks” were previously downplayed within clinical care (while at the same time conflating social difference with health behavior). Providers and staff in this organizational context were thus required to confront issues of gender and sexuality that were previously granted limited attention, with this formal awareness introduced by public reporting requirements and data standardization.

Such data thus marked a new form of “openness,” described among implementation staff as key for promoting a “welcoming environment,” and further noted by the same CCRN:

Interviewer: Do you think it’s important to collect information on SOGI?

CCRN: Oh yes, yes yes. Because we have patients of all cultures and beliefs, and we have to be open, open to whatever. It’s coming, and this really is a major change for us. Especially since I’ve been in the nursing field for 30 years [at County Health System for majority of career] … but when I see patients now, I ask them because we can no longer assume [SOGI]. We must respect that. It’s the patient’s choice.

Asking questions about SOGI was seen as a major change from typical approaches which presumed heterosexuality among patients, instilling a baseline awareness that employees should no longer assume all patients are straight and cisgender. Interviewees thus framed data as drawing employee attention to SGM domains, fostering a formalized awareness against a heteronormative backdrop.

**At LGBTQ Health Center**, in contrast, employees grappled with the advent of SOGI data collection within a context dedicated to addressing LGBTQ health disparities through tailored, affirmative care provision. Providers understood gender and sexuality to be diverse and fluid, and cited not making assumptions about patient sexual and gender identities as foundational to providing affirming care to LGBTQ people (e.g., by asking patients gender pronouns). Through their experiences of caring for SGM patients, providers developed ways to ask gender and sexuality-related questions to affirm care recipients as well as build rapport and trust (Paine, 2021a), but these oftentimes conflicted with the standard SOGI items. Ensuring that patient SOGI data fields were filled out within EHRs was largely seen as a box to be checked that intruded upon time to provide quality care, as one staff member put it:

There’s absolutely cultural resistance [from clinic staff], there’s a lot of, you could say trauma, on behalf of providers. Every time something new rolls out [including SOGI], the answer was, “You have to click here,” and not so much, “This is how the system will help facilitate your work in meeting these measures,” but more, “This is how you’re going to help the system understand you’ve done this [asked about SOGI].”

Another provider confirmed efforts to obtain SOGI data privileged the mandate over actual care delivery designed to improve LGBTQ patient health: “It’s not Patient-Centered, it’s Data-Centered – it’s not really looking at a patient as an individual, it wipes out individuality. And at the Center that’s a huge frustration because our patients are incredibly diverse and different and each one of them needs really different things.” Mandated questions were often understood by providers to be misinformed or even harmful to patient care, given their inability to account for diversity *among* SGM patients (see sections below).

Administrators, HIT staff, and providers at LGBTQ Health Center had also worked to document and address patient needs within EHRs years *before* the SOGI mandate, and the standard questions often conflicted with employees’ preferred approaches to capturing patient data. Long-tenured HIT employees reported advocating for many years to make EHRs and data structuring more inclusive and less determined by dominant gender ideologies, presenting a keen awareness of the importance of data collection for caring for SGM patients. Yet they also understood workflows based on normative assumptions about gender and sexuality (including those embedded within standard SOGI items) to hinder patient care, and continually created workarounds so that providers could perform exams or tests based on anatomy, not gender. Providers therefore understood EHRs to be key to providing affirming care, yet viewed SOGI data mandates as constraining their own established in-house work. Recognizing themselves as leaders in the field, the sudden expectation of reporting SOGI data in effort to redress SGM inequities, after facing resistance from public agencies for so many years, was thus perceived as offensive:

For years we’d say to the CDC, where do trans women go, and they’d say male, and we’d be – that’s fucked up, because they’re not… And it did change, so we sort of function as an informant. I think that’s part of our responsibility to the larger structure, to say, “We know this better than you.” You look at EHRs now in the next session everyone has to capture SOGI data, but for years, we didn’t upgrade because we didn’t have any place to put trans patients that felt even remotely respectful… And now suddenly everyone has to have SOGI data, and being at risk of losing grants because you can’t meet requirements because they’ re dumb.

As noted in the employee’s explanation above, “we know this better than you” reflects a break from the "counting as means of ending LGBTQ invisibility" suggested by data advocates, with organizational informality in this context perceived to better serve the needs of SGM patients. In the context of LGBTQ Health Center, providers experienced at addressing SGM health inequities through tailored care provision balked at the insistence that they change their tried-and-true approaches to capturing SOGI-related information interpreted to be meaningful for care in order to appease reporting mandates. Formalized awareness to issues of gender and sexuality via data standardization thus simultaneously promoted “openness” in a heteronormative environment *while also* “checkboxing” SGM diversity within an LGBTQ setting, with technical standards taking on particular significance across unequal contexts.

### 3.2. Data as bounded appropriateness

Across sites, SOGI data were also viewed through the lens of *bounded appropriateness*—encompassing the limits of what might be acceptable to consider in one setting, while serving as a poor starting point for recognizing marginalized identities and embodiments in another. These differences patterned provider and staff reactions to SOGI data, with workers anticipating different patient expectations of discussing gender and sexuality as shaped by heterogenous contexts.

**At County Health System**, providers and staff often struggled to recognize SOGI data’s relevance for care, further informed by anticipation of patient expectations when discussing gender and sexuality. Despite acknowledgement of “openness,” several health care workers still expressed discomfort around asking patients the SOGI items, suggesting the questions broached a previously unacknowledged boundary of topics appropriate for clinical discussion (Cruz, 2020). Patients typically did not expect to discuss such issues when seeking care in this clinical setting, as described by one staff member:

Licensed Vocational Nurse: Asking about sexual orientation, I’ve got to say, that’s the main one I’ve had challenges with. Our patients really don’t like discussing that because they feel like it’s not necessary for them to notify their physician or clinical staff.

Interviewer: What are your thoughts on that?

LVN: Umm, I feel a bit awkward about it. I really don’t feel comfortable asking them. I try to give them a heads up, “I’m going to ask you about sexual orientation, you’re welcome to decline the questionnaire – you don’t have to answer it.” I automatically put that in place so that they know they don’t have to answer it. But if they say yes, then I’ll read off the questionnaire for them … Sometimes they just decline it, but then they give you that look, “Why do you need to know that?”

A nurse manager also relayed this sentiment, suggesting patients at County Health System questioned why issues of gender and sexuality surface within clinical care, and why SOGI was just now attracting attention:

Some patients laugh [when asked SOGI], like, “I’ve been here for 30 years, why are you asking me these questions now?” “Do you really want to know?” But you just explain, “We’ re doing this thing now…” We don’t force patients to answer if they don’t want to, but sometimes I ask the patient in a joking manner like, “I know right now, you were born as a female, to this day you’re still a female. Could you tell me, are you married? Is it a guy or woman?”… You want to frame it so it is non-threatening but at the same time you ask the questions truthfully and the patient will answer truthfully… It’s really a matter of framing the questions for the patient.

The need to provide framing, here drawing from heteronormative expectations of patients having a current gender identity aligned with assigned sex at birth and presently married, speaks to the contextualizing work providers and staff carry out to collect SOGI data. This is of further significance given the felt risk of stigma and discrimination that may take place within this context, which some providers and staff recognized patients may anticipate encountering at this site of care.

While some providers and staff drew on standard questionnaire instructions and heteronormative assumptions in asking patient SOGI questions, others attributed the SOGI initiative to technology itself in justifying data collection, as noted by the provider below:

Some patients have been like, "What are these questions?" But for a lot of them – I advise my LVN to [say], “We’ve updated the computer system – we’re collecting this new data now and we’re asking everybody this,” and it’s nice that it’s only one time per person that we don’t have to ask it over and over again. I haven’t heard any direct complaints myself, I’ve just heard from an LVN who said, "Yeah, that patient thought it was a little strange.” I’m glad we did not have to go with all the more specific questions about what body parts do you have, because that would have been a little more intrusive, right? I think our patients would have been a little more (*side look*) with that, but so far so good.

Relying on general attributions to “the computer system” and formal data collection procedures, in this context, allows for providers and staff to broach subjects of gender and sexuality when patients otherwise may fear stigma and discrimination. The boundaries imposed by these same standards – such as the one-time collection of items on gender and sexuality – are seen as the limits of acceptability for patients in this particular setting, rather than constraining the very gender and sexual diversity that is readily recognized and centered within LGBTQ organizations.

**At LGBTQ Health Center**, in contrast, these same questions served as a poor starting point for discussing gender and sexuality in relation to care. Indeed, in the context of LGBTQ care provision, SOGI data were seen as *inappropriate* for the very reasons the technical standards were potentially acceptable in other clinical contexts—namely, their bounded treatment of gender and sexual diversity. Employees did emphasize the importance of asking SGM patients affirming questions; however, their understanding of how to do so among majority LGBTQ patients typically diverged from the data mandate. The SOGI questions were instead perceived to detract from one’s ability to appropriately care for SGM patients, as one provider put it:

I don’t think we’ve found necessarily the right way to make sure the EHR [and SOGI questions don’t] feel intrusive during the patient visit, to make it seem like it’s not a secretive document that I’m writing that’s mine as a medical provider, and even though it’s about you, you can’t see it. And we have not done a good job of using health information technology [and mandated data collection] to facilitate communication with our patients.

Instead, providers learned to use multiple data fields to facilitate their work, without consideration of standard collection of SOGI for reporting purposes. One said: “I’ve learned to document the most important things. Some of it I leave clear, and I go back when I leave the patient. I don’t want to be in front of somebody and fill it out. I think it distracts… I think it causes more problems than it’s helpful…” Providers and staff also recognized the standard SOGI items were unable to capture the fluidity of gender and sexuality over the life course, nor did they capture differences in body parts *within* social categories, key issues for the provision of optimal care for gender and sexually diverse patients. This marked a strong contrast from County Health System, where the normative bounds inherent in standard items touched the limits of acceptability, which instead provoked frustration among those with greater experience working with diverse SGM patients at LGBTQ Health Center.

Providers also recognized that SGM patients may have previously encountered stigmatization and misrecognition in majority medical settings, and insisted that patients should be met “where they are at” when discussing gender and sexuality, especially in caring for trans and nonbinary patients. When asked what this provider did to affirm patients, they continued:

I think with the specifics of working with trans patients, not assuming a gender trajectory, and asking a lot of questions in a sensitive way. I think it’s important to approach things with curiosity in mind, and not assuming any standard transition, whether its social, hormonal, emotional, spiritual, *it’s different for every single person*… I try to keep that in mind and be curious about that, not only asking the questions, but asking them in a way that meets the patient where they are. Some people are really into discussing nuances, and *want* to be asked. So it’s about respecting what people want to tell you. They can tell you as much as they want.

Providers at this site, therefore, also expressed concern about how SOGI-related questions impact patients, but from the opposite perspective: that mandated SOGI questions hinder patient-centered open-ended discussions that affirm marginalized gender and sexual identities and embodiments, and that standard data mandates therefore do not necessarily prioritize LGBTQ well-being. Thus both sites viewed the SOGI data as bounded in appropriateness, with unequal contexts shaping the extent to which boundaries are understood to facilitate or hinder care.

### 3.3. Data as limited information

Advocates and public officials suggest SOGI data can assist providers and staff in providing population-specific treatment based on patient gender and sexuality, yet this argument obscures the fundamental importance of background knowledge on SGM health for providing good care. Providers and staff at both sites confronted the SOGI data as *limited information*, in one context because of lack of understanding of health inequities in justifying data items, and the other because of data’s inability to stand in for acquired knowledge and experience.

**At County Health System**, standardized SOGI data did introduce initial awareness of marginalized identities and embodiments in a heteronormative environment, seemingly promoting attention to non-heteronormative forms of gender and sexuality. Despite the benefits such data appear to offer, however, the initiative ultimately failed to provoke a deep understanding of SGM health concerns, with patient differences acknowledged yet rarely connected to the problem of inequity. A staff member involved in the initiative expressed frustration that providers and staff often did not view SOGI data through a structural lens, nor did they have the support to develop this understanding given prior training and time constraints (Cruz, 2020). She suggested data mandates do not guarantee work towards redressing inequity, comparing her experience in HIV/AIDS advocacy with primary care:

It’s been challenging. You just can’t teach cultural competency [on SOGI] or eliminate stigma in a 40-min presentation – it’s just not going to happen no matter how great your presentation is. It takes time for people to change or to look at things in a different way, and we’re now asking people to make change quickly. So that’s the challenge – how do we get people’s buy-in knowing that we’re giving them a short timeframe to do this [collect data]?

Without key background knowledge of social determinants and health disparities, providers and staff were less inclined to buy into the initiative and incorporate standard data within care practices, let alone approach their work as an avenue for reducing inequity. This same program manager relied on case studies to communicate to providers and staff, yet recognized this was a limited means of conveying the structural nature of SGM health inequities:

It’s hard – just this week [providers and staff] were asking, “But why is it important that we ask these SOGI questions?” And one of the case studies I used was a trans person – if you don’t ask their sex assigned at birth and gender identity, you might miss a screening for breast cancer. If they just say “male,” you’re not going to catch that… I think that’s a peek into what a disparity would look like, but not really, so that’s the challenge – how do I explain [SGM inequities] in a quick way to somebody who doesn’t even have a concept of this?

In this particular context, the data thus provide *limited information* in that they are unable to capture the more fundamental issue at stake: provider, staff, and administrator lack of knowledge and experience in working with SGM populations, and lack of understanding of how such work contributes to broader objectives of reducing health inequities.

As a result, even providers and staff who expressed support for the SOGI initiative did not always appreciate the social significance of data collection or its implications for patient care. Despite some employees recognizing SOGI data as key for respecting patient differences, this recognition often failed to consider population-based forms of treatment as suggested by public officials and data advocates. And as important as respectful interactions are for SGM care, respect alone cannot stand in for domain-specific knowledge (such as the distinction between SO and GI, and the multiple dimensions of identity, behavior, and embodiment) and acquired clinical experience working with diverse SGM patients. A nurse manager similarly suggested most staff lack in-depth understanding of SOGI’s relevance for tailored care provision:

Sexual orientation, it’s a very difficult item to ask, but first you have to understand why you are asking it. It’s important for staff to understand why they are asking all of these questions in order to explain it to the patient. It’s not to shame you, or “the norm is this and you are this,” it has absolutely nothing to do with that… it’s just to understand to take care of you as a person… so I get it, but is it a difficult question to ask? Yes, yes it is.

This lack of understanding is informed by institutional heteronormativity, given the former absence of recognizing SGM patients and their health needs within routine system proceedings. When combined with time constraints and other reporting requirements, this particular context resulted in providers and staff mandated to collect data but with uneven understanding of why such data were important, yielding limited information to redress inequity.

**At LGBTQ Health Center**, however, providers saw themselves as advocates and allies—as well as often being SGM themselves—in improving the health of LGBTQ people, and understood themselves to be doing the work of reducing SGM health inequities directly. As one shared, “I take care of HIV positive folks and trans folks, and a lot of them have been traumatized by the [medical] system. I deal with a lot of people who have been traumatized, so I feel like a lot of my work is just convincing people to stay in care, and to like actually engage.” Providers and staff also reported learning more about gender, sexuality, and health on the job, but many arrived at the organization with background knowledge of social determinants and health disparities and strong dedication to redressing factors undermining the health of LGBTQ people. As such, they were experienced with asking questions about gender and sexuality—yet their experiences had taught them that SGM identities and behaviors are not static, and therefore efforts to capture patient data on SOGI are not necessarily clinically useful (Paine, 2021b). And although some providers expressed concern over how to train employees about social determinants, they also emphasized developing a structural understanding of health inequities develops over time through hands-on experience with caring for SGM patients. Reflecting on their experience, one provider suggested:

[You want to] try not to be so “sensitive” that you’re like, “Are you a lesbian?”… [But rather] “I’m not going to assign things to you, even in the name of sensitivity,” – because as you are well aware, you’re frequently wrong when you make assumptions, even if they’re sensitive ones. So, I think it’s really important to just say, tell me about yourself, but conveying that you’re open to hearing any answer… I think it’s really important to encourage self-definition.

Although providers had bought in to the importance of creating care landscapes and approaches that address social determinants, including understanding patient sexuality and gender, their frameworks differed from those imposed by SOGI reporting mandates and instead drew from their own experiences. Given employee positions as advocates and experts in the provision of affirming care, data monitoring was thus understood as less important than personalized care.

Employees further connected this to the evolution of reporting requirements over time, including the burden placed onto providers due to changes in data collection protocols, which generated pushback due to the limited scope of resulting information. As one administrator put it:

There’s very specific kinds of data you need to collect, like certain things you need to collect and you need to do it in a certain way. It’s not so much the *things you need to do, or things you want to do for your patients* [emphasis added, “good care”]. I think [SOGI reporting] is so specific as to how you have to collect the data, that it’s been a hard change, and some providers are like, “It’s too many clicks, I’m not going to do it, I’m just going to type it in this box here.” But then it doesn’t count [for reporting]… I think the tension always exists between the bean counters and bean cookers, whatever they are, bean growers, maybe.

As “bean growers,” these providers understood themselves as distinct from organizations that perpetuate stigma and marginalization, or which lack understanding of disparities: instead, they viewed their work as centrally involved in redressing inequity *over and beyond* standard data mandates. Data collection, as a technique of quantifying gender and sexual difference for reporting purposes, is positioned to be in direct tension with providing good care itself, shifting focus away from patient care and towards EHR documentation for public reporting. Data-centered initiatives thus offer limited information to providers and staff in caring for SGM patients, obscuring the importance of background knowledge, experience, and commitment in redressing inequity.

## 4. Discussion

Data infrastructures created by EHRs and mandated reporting are commonly framed as offering new opportunity to address fundamental causes and reduce health disparities, linking EHR data sources to broader objectives of reducing inequality (Institute of Medicine, 2014; Adler and Stead, 2015; Douglas et al., 2015). As health systems face growing pressure to quantify health outcomes, costs, and equity goals via performance metrics from EHR data sources, standardized data is expected to expand public visibility of clinical encounters, inform state programs of disparity reduction, and induce providers and staff to redress social problems through clinical care (Institute of Medicine, 2013; Anderson et al., 2018; DeMeester et al., 2017; Cantor and Thorpe, 2018). The stakes of quantification are therefore quite high: without measuring “the social,” advocates and experts warn, biomedicine will continue to elide social factors driving health outcomes, thereby reproducing health inequalities (Cahill and Makadon, 2013; Douglas et al., 2015; Penman-Aguilar et al., 2016; Zhang et al., 2017; Wasserman et al., 2019). At the same time, public anxieties over the rise of “data-driven care” suggest potential structural limitations with data-centered approaches, including deeper concern over what data standardization captures and what it obscures (Cruz, 2020; Thompson, 2020).

Our social scientific findings—drawn from comparative qualitative fieldwork across two starkly different sites of care navigating the same data mandate—reveal how the social problem of inequity varies considerably across clinical contexts, informed by institutional conditions that transcend point of care data collection. We find standard data may promote visibility of “social factors” in certain clinical settings, yet can also constrain clinical utility among providers and staff with greater knowledge and experience with related domains (Gaspar et al., 2020; Thompson, 2020). We further illustrate how data reporting may result in limited attenuation of health inequities, despite broad expectations of clinical change (Institute of Medicine, 2014: 17; Douglas et al., 2015; Zhang et al., 2017): evidence from our two sites illuminates how entrenched inequities actively inform organizational asymmetries in serving different communities (Paine, 2021a, 2021b; Martos et al., 2017; Ingraham and Rodriguez, 2021), discussing related topics with patients as a part of care (Cruz, 2020; Carabez et al., 2015; Banerjee et al., 2018), and establishing provider and staff knowledge and experience in redressing social problems (Donald et al., 2017; Mansh et al., 2015). Our fieldwork “on the inside” of clinical settings thus reveals a much more fundamental set of stakes at play with data standardization, beyond implementation and associated technical challenges (Cruz, 2021). By leveraging qualitative social science methods, our findings suggest that the same data-centered strategies that prioritize *capturing patients* simultaneously miss the *unequal contexts* of care, with standard data highlighting certain elements of inequity at the direct expense of others.

While universal technical standards are expected to unify disparate sites of care to produce aggregate data, we further show how common standards serve as points of convergence *and* divergence across social contexts (Bowker and Star, 1999; Timmermans and Epstein, 2010). Standard data on “social factors” serve as *formalized awareness*, recognizing marginalized identities and embodiments but checkboxing diverse needs; *bounded appropriateness*, introducing notification by foreclosing open-ended discussion; and *limited information*, shaping what can be known while obscuring the social foundations of good care. By presenting these three lenses, we have also demonstrated how providers and staff *already* work to provide care “in context” across diverse settings—with this context itself reflecting biomedical stratification that remains unlikely to transform via data standardization alone. Providers and staff continuously created workarounds to appease reporting requirements, oftentimes tinkering with standard protocols *in spite* of common data mandates, rather than because of them (Thompson-Lastad and Rubin, 2020; Timmermans and Berg, 2003).

This is not to suggest that standardized data have no role whatsoever in the governance of social problems: clearly, measuring the social world into discrete categories is useful for monitoring changes over time, comparing across sites, and evaluating differences within settings. But to solely collect data on patient social factors, including within EHR data sources, is to locate inequity as everywhere among *patients* but nowhere within *institutions*, capturing social identities while fundamentally missing the social context of biomedical stratification. These issues are by no means limited to SOGI data specifically or SGM health inequities: expanded investment in data-centered approaches within biomedicine touches on far-reaching social problems, including housing and food insecurity, education, employment, and race and ethnicity (Table 1; Institute of Medicine, 2014; Cantor and Thorpe, 2018; NASDOH 2019; Douglas et al., 2015; Wasserman et al., 2019). In presenting a comparative case study of data-driven care, we unveil the deeply entrenched nature of biomedical stratification that continues to elude patient-level measurement and data standardization, joining other critical data scholars to call for deeper engagement with the social context of inequality (Benjamin, 2019; Noble, 2018; Thompson, 2020; Westbrook, Budnick, and Saperstein 2021).

## 5. Conclusion

In the era of data analytics, where clinical algorithms, data dashboards, and other high-tech innovations reign supreme across institutional spheres, data collection on “social factors” may at first glance appear key to redressing the social basis of health inequities. But we insist that data-intensive approaches alone, as useful as they may be for population-level comparison, remain unable to account for the complex dynamics of enduring social problems, and ill-designed to redress them. While recognizing advocate and expert arguments for quantifying “the social” against the general swell of data-driven care, we challenge all audiences to engage with a broader range of expertise in addressing social determinants and health inequality. In this article, we have demonstrated how qualitative sociological research on the integration of standard data within clinical settings reveals contextual factors that shape the very problem data collection may *appear* to redress, yet that ultimately remain unchanged given the powerful yet unmeasured social context of inequity. We suggest addressing “social factors” within clinical care will require more than public investment in data-driven interventions: it will require contextualizing enduring stratification within biomedicine itself, while confronting the relentless imperative to quantify in lieu of fundamental social transformation.

## Figures and Tables

**Table 1:** Social and Behavioral Domains for Inclusion in EHRs.

**Sociodemographic Domains** Sexual orientation Race/ethnicity Country of origin Education Employment Financial resource strain (Food and housing insecurity)
**Psychological Domains** Health literacy Stress Negative mood and affect (Depression, anxiety) Psychological assets (Patient engagement/activation, self-efficacy)
**Behavioral Domains** Dietary patterns Physical activity Tobacco use and exposure Alcohol use
Individual-Level Social Relationships Domains Social connections and social isolation Exposure to violence

**Source:** Institute of Medicine (2014). Capturing Social and Behavioral Domains in EHRs: Phase 1.

**Table 2:** HRSA UDS Demographic Reporting on SOGI.

Patients by Sexual Orientation	Patients by Gender Identity
Lesbian or gay	Male
Straight (not lesbian or gay)	Female
Bisexual	Transgender Male/FTM
Something else	Transgender Female/MTF
Don’t know	Other
Choose not to disclose	Choose not to disclose

**Source:** HRSA Program Assistance Letter (PAL 2016-02). “Approved Uniform Data System Changes for Calendar Year 2016.”

**Note:** The UDS demographic reporting mandate on SOGI includes items on patient sexual and gender identity. While both scholars and measurement working groups note the multiple dimensions of gender and sexuality, including identity, attraction, behavior, and embodiment (SMART 2009; FIWG 2016), the reporting mandate prioritizes population-identifying items given program focus on disparity monitoring. We note that the limited focus on patient identity as a means of assessing social difference fails to capture the full prism of gender and sexuality, including as these domains relate to the dynamics of stratification and associated inequity (Westbrook, Budnick, and Saperstein 2021; Cruz, 2017; Paine, 2018).

**Table 3:** Standard data across unequal contexts.

Data on “social factors” as …	County Health System	LGBTQ Health Center
**Formalized awareness**	“Openness”: SOGI questions as formally highlighting attention to non-heteronormative gender and sexuality	“Checkboxing”: SOGI questions as constraining gender and sexual diversity via standard response items
**Bounded appropriateness**	Standard SOGI items as “the limit” of appropriateness; providers and patients do not expect discussion within care	SOGI items as “poor starting point”; providers and patients expect open-ended discussion on gender and sexuality
**Limited information**	Provider, staff lack essential knowledge, experience, and understanding of “good care” — data as weak substitute	Provider, staff possess strong knowledge, experience, and understanding of “good care”— data as interference
